# Ibrutinib plus CIT for R/R mature B-NHL in children (SPARKLE trial): initial safety, pharmacokinetics, and efficacy

**DOI:** 10.1038/s41375-020-0749-5

**Published:** 2020-02-18

**Authors:** G. A. Amos Burke, Auke Beishuizen, Deepa Bhojwani, Birgit Burkhardt, Véronique Minard-Colin, Robin E. Norris, Edita Kabickova, F. Guclu Pinarli, Nurdan Tacyildiz, Angela Howes, Jan de Jong, Grace Liu, Kerri Nottage, Mariya Salman, Xavier Woot de Trixhe, Mitchell Cairo

**Affiliations:** 1 Department of Paediatric Haematology, Oncology and Palliative Care, Cambridge University Hospitals NHS Foundation Trust, Addenbrooke’s Hospital, Cambridge, UK; 2 Department of Hematology and Oncology, Princess Máxima Center for Pediatric Oncology, Utrecht, Netherlands; 3 Children’s Center for Cancer and Blood Diseases, Children’s Hospital Los Angeles, University of Southern California, Keck School of Medicine, Los Angeles, CA USA; 4 Pediatric Hematology and Oncology, University Hospital Münster, Münster, Germany; 5 Department of Child and Adolescent Cancer, Gustave Roussy, Paris, France; 6 Department of Pediatrics, University of Cincinnati College of Medicine, Cincinnati Children’s Hospital Medical Center, Cincinnati, OH USA; 7 Department of Pediatric Hematology and Oncology, Charles University and University Hospital Motol, Prague, Czech Republic; 8 Department of Pediatric Oncology, Gazi University, Ankara, Turkey; 9 Department of Pediatric Hematology and Oncology, Ankara University, Ankara, Turkey; 10 Clinical Oncology, Janssen R&D LLC, High Wycombe, UK; 11 Clinical Pharmacology, Janssen R&D LLC, San Diego, CA USA; 12 Clinical Oncology, Janssen R&D LLC, Raritan, NJ USA; 13 Pharmacometrics, Janssen R&D LLC, Beerse, Belgium; 14 Department of Pediatrics, Medicine, Pathology, Microbiology and Immunology and Cell Biology and Anatomy, New York Medical College, Valhalla, NY USA

**Keywords:** Drug development, B-cell lymphoma

Pediatric patients with relapsed/refractory (R/R) mature B-cell non-Hodgkin lymphoma (B-NHL) have a 2-year overall survival rate with chemoimmunotherapy (CIT) of 15–33% [1, 2]. Rituximab plus ifosfamide, carboplatin, and etoposide (RICE) is widely used in R/R children with NHL [3] and rituximab plus vincristine, ifosfamide, carboplatin, idarubicin, and dexamethasone (RVICI) has been used in Europe [4, 5].

In preclinical studies, ibrutinib, a Bruton’s tyrosine kinase inhibitor approved to treat adults with various B-cell malignancies in the United States and the European Union, among other countries [6], inhibited Burkitt lymphoma (BL; the predominant pediatric mature B-NHL) and diffuse large B-cell lymphoma (DLBCL) tumor cell growth, and prolonged survival in BL xenografted mice [7–10].

We report safety, pharmacokinetics, and preliminary efficacy findings from the run-in stage (part 1; December 2016–December 2018) of an ongoing phase 3 trial (SPARKLE).

Of 21 patients with mature B-NHL (median age, 8 years [range, 3–17]; Table 1), 11 received ibrutinib plus modified RICE (RICE modified with dexamethasone) and 10 received ibrutinib plus RVICI (Supplementary Fig. 1). Detailed methods are presented in the Supplementary Appendix, and Supplementary Tables 1 and 2 show the dosing and administration schedule for these treatment regimens. In ibrutinib plus modified RICE and ibrutinib plus RVICI groups, respectively, zero and four (40.0%) patients had central nervous system (CNS) disease, three (27.3%) and four (40.0%) had bone marrow involvement, and one (9.1%) and six (60.0%) received >1 prior line of therapy (>1 relapse).

**Table 1:** Patient demographics and baseline characteristics.

	Ibrutinib plus modified RICE (*n* = 11)	Ibrutinib plus RVICI (*n* = 10)
Median age (range), years	11.0 (3–17)	8.0 (4–15)
Age group, years, *n* (%)		
1–5	2 (18.2)	2 (20.0)
6–11	4 (36.4)	6 (60.0)
12–17	5 (45.5)	2 (20.0)
Sex, *n* (%)		
Female	3 (27.3)	1 (10.0)
Male	8 (72.7)	9 (90.0)
Ethnicity, *n* (%)		
Hispanic or Latino	2 (18.2)	0
Not Hispanic or Latino	6 (54.5)	9 (90.0)
Unknown	1 (9.1)	0
Not reported	2 (18.2)	1 (10.0)
Race, *n* (%)		
White	9 (81.8)	10 (100.0)
Asian	1 (9.1)	0
Not reported	1 (9.1)	0
Time from initial diagnosis to first dose of study drug (months)		
Mean (SD)	11.7 (10.94)	10.5 (6.22)
Median (range)	7.0 (4–37)	8.2 (5–25)
Type of mature B-cell NHL at initial diagnosis, *n* (%)		
Burkitt-like lymphoma	2 (18.2)	1 (10.0)
Burkitt lymphoma	4 (36.4)	3 (30.0)
Burkitt leukemia	2 (18.2)	4 (40.0)
Diffuse large B-cell lymphoma	3 (27.3)	0
Primary mediastinal B-cell lymphoma	0	1 (10.0)
High-grade B-cell lymphoma	0	1 (10.0)
Other	0	0
Extra nodal sites, *n* (%)		
Central nervous system	0	4 (40.0)
Bone marrow	3 (27.3)	4 (40.0)
Other	9 (81.8)	9 (90.0)
Prior lines of therapy, *n* (%)		
1	10 (90.9)	4 (40.0)
>1	1 (9.1)	6 (60.0)

*NHL* non-Hodgkin lymphoma, *SD* standard deviation, *modified RICE* rituximab, ifosfamide, carboplatin, and etoposide, with the addition of dexamethasone, *RVICI* vincristine, ifosfamide, carboplatin, idarubicin, that includes dexamethasone.

In ibrutinib plus modified RICE and ibrutinib plus RVICI groups, respectively, median number of treatment cycles was 3.0 (range, 1–4) and 2.0 (range, 1–4), and eight and three patients completed ≥3 cycles (Supplementary Fig. 1).

Despite limitations of cross-trial comparisons, including small patient numbers and trial design differences, safety of combined therapies was consistent with known ibrutinib, RVICI, or RICE safety profiles and experience with ibrutinib plus RICE or rituximab, cyclophosphamide, doxorubicin, vincristine, and prednisone in adults with DLBCL [11]. All patients had 1 or more grade ≥3 treatment-emergent adverse events (TEAEs), with >50% of patients in either group having hematologic, gastrointestinal, infectious, and metabolism- or nutrition-associated events (Supplementary Table 3). Ten (90.9%) patients in ibrutinib plus modified RICE and nine (90.0%) in ibrutinib plus RVICI groups had serious TEAEs (all grade ≥3). Most frequent grade ≥3 ibrutinib-related TEAEs (i.e., those considered related by the investigator) were thrombocytopenia (42.9%), neutropenia (38.1%), anemia (33.3%), and febrile neutropenia (19.0%; Supplementary Table 4).

Two patients in ibrutinib plus modified RICE and three in ibrutinib plus RVICI groups had major hemorrhage events (grade 3/4; in the setting of thrombocytopenia; Supplementary Table 5). Two major hemorrhage events (intestinal and intracranial) in the ibrutinib plus RVICI arm were considered ibrutinib related. Major hemorrhage has occurred in 4% of 2838 ibrutinib-exposed patients in 27 previous clinical trials [12].

Hematologic TEAEs were expected, particularly with background CIT. In a study of 20 children with R/R B-NHL receiving RICE alone, rituximab-related AEs, infections, and hematologic toxicities (e.g., neutrophil- and platelet-related events) occurred frequently, and one child discontinued because of prolonged myelosuppression [1].

As expected with the CIT regimen [1], bone marrow suppression, reported as laboratory abnormality of low hemoglobin, platelets, or neutrophils, was common (Supplementary Appendix). Hematologic events were generally manageable, most recovering before the next cycle without delaying treatment.

**Fig. 1:**
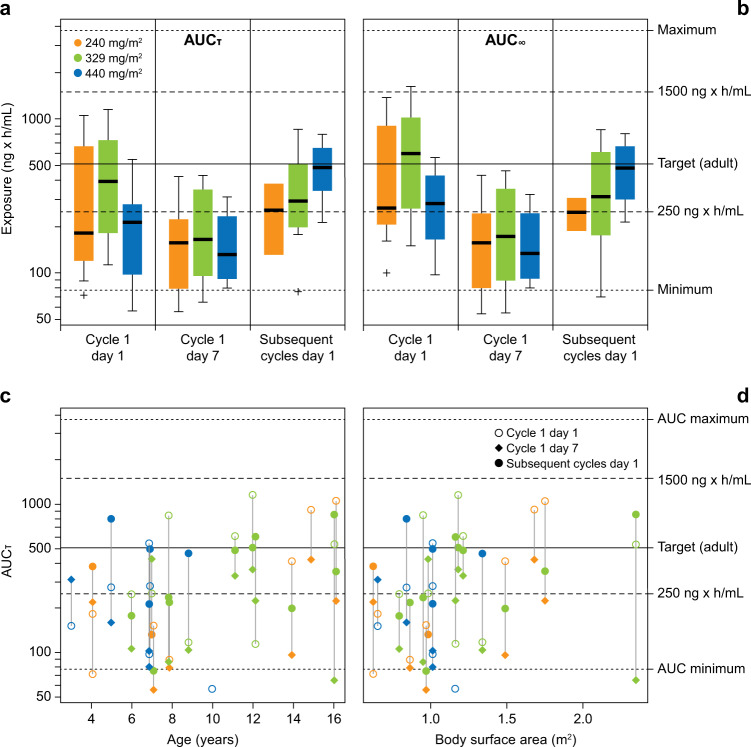
Ibrutinib exposures with 240–440 mg/m^2^/day doses. Box/whisker plots of estimated AUC on pharmacokinetic occasions by cycle and day for each dose for (**a**) AUC_τ_ (estimated AUC of 24-h dosing interval) and (**b**) AUC_∞_ (dose*F/CL; predicted AUC at steady state). Solid line represents median, box represents 25/75%, and whiskers represent 10/90% confidence interval. Individual symbols represent outliers. (**c**) AUC_τ_ versus age and (**d**) versus body surface area. Vertical lines represent individual patients, color represents dose, and symbol represents pharmacokinetic occasion. Target AUC range based on adult exposures was 250–1500 ng × h/mL. *AUC* area under the plasma concentration–time curve.

Pharmacokinetic data were assessed for <12 years and ≥12 years. For patients aged 12–17 years (*n* = 6), area under plasma concentration–time curves (AUCs) were within target range (250–1500 ng × h/mL), supporting the 329 mg/m^2^/day dose (not exceeding 560 mg daily). However, in younger patients (*n* = 14), AUCs at 329 mg/m^2^/day were lower than expected, and 440 mg/m^2^/day was required to obtain exposures mostly within target range (Fig. 1). No AUC values were >1500 ng × h/mL regardless of age or dose. AUC value on cycle 1 day 7 was notably lower than day 1.

Although treatment was scheduled to avoid overlapping ibrutinib and mesna dosing, this was not always possible given prolonged mesna infusion during days 3–9 with RVICI. Mass spectrometric and radiometric analysis of 14C-labeled ibrutinib and mesna, or ifosfamide with metabolizing enzymes, did not show ibrutinib adduct formation or decreased ibrutinib concentrations. Inter- and intraindividual AUC value variability occurred and exceeded variability in adults. Inter-sample discrepancies were potentially introduced by cytochrome P450 subtype 3A inhibitors/inducers (one patient used CYP3A inhibitor, one used CYP3A inducer) before/during ibrutinib treatment (considered a study protocol deviation), ibrutinib administration via nasogastric tube in some patients, or time and content of food intake differences pre- and post-ibrutinib administration.

Physiology-based pharmacokinetic simulations using an adult ibrutinib model [13] suggested body surface area–based dosing would result in adult range exposures (Loeckie de Zwart, May 28, 2015, unpublished data). Although the youngest patient was almost 4 years old, a predicted increase in patients aged <6 years was uncorroborated. Exposures were still at the low end of the target range, but above observed minimum exposure in adults at 560 mg/day (shown as efficacious in adult B-cell malignancies).

In the ibrutinib plus modified RICE group, there were three progressive disease (PD)-related deaths (one of which was due to multiple organ failure within 30 days of the last dose) and one AE (septic shock)-related death within 30 days of the last dose occurred. In the ibrutinib plus RVICI group, three TEAE-related deaths (unrelated to ibrutinib; within 30 days of last dose; two due to sepsis and one because of neutropenic sepsis) and six PD-related deaths occurred. Most of these patients had multiply relapsed disease and were heavily pretreated pre-enrollment; therefore, they had poor hematologic reserve and were highly susceptible to complications due to therapy.

Preliminary efficacy findings were promising. As prognosis for this pediatric patient population is poor, achieving a complete response (CR) to proceed to high-dose consolidative therapy is important [1]. Of 21 patients who received ≥1 dose of ibrutinib, 12 (57.1%) experienced a response. In the ibrutinib plus modified RICE group, eight (72.7%) were responders, among whom three (27.3%) had a CR, including one unconfirmed CR (one DLBCL, one BL, one Burkitt leukemia [B-AL]), and five (45.5%) had a partial response (PR; one BL, two DLBCL, two Burkitt-like lymphoma). In the ibrutinib plus RVICI group, four (40.0%) were responders, among whom two (20%) had a CR (both B-AL) and two had a PR (one B-AL, one high-grade B-cell lymphoma). Across both groups, all responders received treatment at first relapse, except one with high-grade B-cell lymphoma in the ibrutinib plus RVICI arm who achieved PR in second relapse.

Among 14 patients treated after first relapse, 11 (78.6%) were responders (five CR, six PR), and one of seven (14.3%) treated after second relapse was a responder (one PR). Supplementary Table 6 presents response by histology. Although this part of the study was not designed to compare efficacy of either regimen, overall response and CR rates were higher with ibrutinib plus RICE than ibrutinib plus RVICI. At an 18-month median follow-up, median investigator-assessed event-free survival (EFS) was unreached in the ibrutinib plus modified RICE group and 2.4 months in the ibrutinib plus RVICI group (Supplementary Fig. 2), potentially because more patients treated with the latter had received >1 prior line of therapy. Treatment at first relapse (versus second relapse) was associated with a higher response rate and longer EFS possibly because patients tolerated therapy better and did not discontinue prematurely due to AEs. In addition, unlike those in the ibrutinib plus RICE group, some patients in the ibrutinib plus RVICI arm had CNS involvement, and therefore a poorer overall prognosis. Given the exploratory nature of the EFS analysis and small sample size, it is difficult to ascertain whether the addition of ibrutinib to CIT is beneficial and results should be interpreted with caution.

Seven patients subsequently received hematopoietic stem cell transplantation (HSCT; four in the ibrutinib plus modified RICE group and two in the ibrutinib plus RVICI arm after completing ≥3 treatment cycles, and one after completing two cycles of ibrutinib plus RVICI and one cycle of ibrutinib monotherapy; Supplementary Fig. 1).

Results of part 1 of the trial support the continued assessment of ibrutinib with modified RICE/RVICI in this patient population. Part 2 is assessing the efficacy of ibrutinib (329 mg/m^2^/day in patients aged ≥12 years and 440 mg/m^2^/day in patients aged <12 years) plus modified RICE/RVICI versus modified RICE/RVICI CIT alone in a randomized fashion. Pharmacokinetic sampling in part 2 is on day 15 instead of day 7 of cycle 1 to avoid potential interactions. Because preliminary efficacy findings were better in patients treated upon first relapse, the protocol was amended to only include patients in first relapse. Due to the very poor prognosis for pediatric patients with R/R mature B-NHL, trials such as this, while challenging to conduct, are critically important.

### Supplementary information

Supplementary Information

Supplementary Figure 1

Supplementary Figure 2
