# Supplementary material for: Ibrutinib plus CIT for R/R mature B-NHL in children (SPARKLE trial): initial safety, pharmacokinetics, and efficacy
Source: Leukemia. 2020 Feb 18;34(8):2271–5. doi: 10.1038/s41375-020-0749-5 (PMC7387295; doi:10.1038/s41375-020-0749-5)
Supplement: Supplementary file 1 — Supplementary Information [file 41375_2020_749_MOESM1_ESM.docx]

**Supplementary Information**

**Materials and methods**

The SPARKLE trial (NCT02703272) was approved by the institutional review board or independent ethics committee according to national requirements for recruitment at each participating institution and conducted according to ethical principles defined by the Declaration of Helsinki and the International Conference on Harmonisation Guidelines for Good Clinical Practice. Informed consent and assent where appropriate were obtained before initiating the study.

**Patient eligibility**

Eligible patients were aged 1 to < 18 years with R/R BL, Burkitt-like lymphoma, Burkitt leukemia (B-AL), DLBCL, or another pediatric mature B-NHL, as defined by the 2016 World Health Organization classification [1]. Diseases were required to be in first or later recurrence or primarily refractory to conventional therapy. Diagnoses were assessed and determined locally, and patient histology reports were centrally reviewed by the study responsible physician before screening. Included patients had ≥ 1 site of measurable disease, defined as the presence of lesions > 1 cm in the longest and shortest diameters as measured by radiologic imaging, bone marrow involvement, or cerebrospinal fluid involvement. Additional eligibility criteria included a Lansky-Karnofsky score ≥ 50, an absolute neutrophil count of
≥ 500 cells/µL, and a platelet count of ≥ 50 000 cells/µL (or ≥ 25 000 cells/µL in the event of bone marrow infiltration).

Key exclusion criteria were allogeneic bone marrow transplant within 6 months prior to initiation of treatment, a diagnosis of post-transplant lymphoproliferative disease, ongoing anticoagulation with vitamin K antagonists or an inherited or acquired bleeding disorder, clinically significant arrhythmia, complex congenital heart disease, and left ventricular dysfunction.

**Study design and treatments**

SPARKLE is an ongoing two-part, multicenter, randomized, open-label, phase 3 trial assessing the safety and efficacy of ibrutinib combined with modified RICE (with the addition of dexamethasone) or standard RVICI (which includes dexamethasone) compared with CIT alone in pediatric patients with R/R mature B-NHL. Part 1 was designed to assess safety and pharmacokinetics of ibrutinib as an add-on therapy to modified RICE or RVICI CIT and was conducted across 17 centers in Brazil (one site; one patient), the Czech Republic (one site; two patients), France (three sites; three patients), Germany (three sites; three patients), Italy (two sites; three patients), Korea (one site; one patient), Poland (one site; one patient), Romania (one site; one patient), Turkey (three sites; five patients), and the United States (one site; one patient).

Up to 24 patients were planned for enrollment in Part 1 of the study (non-randomized). Patients were stratified into the age groups of 1–5 years, 6–11 years, and 12–17 years. Although for most histologies investigated in this study, we did not expect a difference in response by histology, to date, there is no experience with ibrutinib in Burkitt lymphoma to indicate whether there will be a difference in response by histology, hence histology was also included as a stratification factor. The evaluated doses of ibrutinib were 240, 329, and 440 mg/m^2^/day (not exceeding 560 mg/day). The first two patients in each age group received a starting dose of 240 mg/m^2^/day (i.e., the approximate 420 mg/day equivalent dose of ibrutinib). If there were no safety concerns and the exposure (area under the plasma concentration–time curve [AUC]) in the first cycle did not exceed the target range, the dose was increased to 329 mg/m^2^/day (i.e., the 560 mg/day equivalent dose of ibrutinib).

Although BTK occupancy was monitored, no specific occupancy was targeted; the assumption instead was that achieving systemic exposures similar to adults would also result in complete occupancy in the investigated pediatric cohort.

Ibrutinib was supplied to patients either as capsules or as a suspension formulation and given in combination with CIT (i.e., the investigator’s choice of modified RICE or RVICI) on a 21- to 28-day treatment cycle for three cycles. A cycle of the modified RICE regimen consisted of 375 mg/m^2^ rituximab on days 1 and 3, 3 g/m^2^ ifosfamide on days 3, 4, and 5, 635 mg/m^2^ carboplatin on day 3, 100 mg/m^2^ etoposide on days 3 through 5, and 20 mg/m^2^/day dexamethasone on days 5 through 9. A cycle of the RVICI regimen consisted of 375 mg/m^2^ rituximab on days 1 and 3, 0.4 mg/m^2^ vincristine on days 3 through 6, 10 mg/m^2^ idarubicin on days 3 and 4, 200 mg/m^2^ carboplatin on days 3 through 6, 2 g/m^2^ ifosfamide on days 3 through 7, and 20 mg/m^2^/day dexamethasone on days 5 through 9.

Ibrutinib was administered as a capsule or suspension, and patients could switch between formulations because treatment complications could lead to difficulty in swallowing capsules, except on days when samples to assess pharmacokinetics were taken. A prior study (NCT02390609) showed that the relative bioavailability of a single-dose of ibrutinib suspension formulation versus capsules (560 mg) was similar with respect to AUC (Jan de Jong, James Jiao, Peter Hellemans, unpublished data, September 29, 2016).

All patients were administered intrathecal methotrexate, corticosteroid, and cytarabine in age-appropriate dosing on day 1 of each cycle for prophylaxis of central nervous system (CNS) disease. Patients with CNS disease were given additional doses of triple intrathecal therapy every 4 days until cerebrospinal fluid was cleared of blasts. During ifosfamide administration, mesna was given to patients at a different time from ibrutinib dosing to avoid potential drug-drug interactions (Supplementary Tables 1 and 2).

Following the completion of three cycles of combination therapy, patients treated with ibrutinib who had demonstrated a partial response (PR) or better continued ibrutinib monotherapy at the same daily dose for three 28-day cycles or until disease progression, unacceptable toxicity, or the start of subsequent antilymphoma therapy or a conditioning regimen for stem cell transplantation.

Part 2 of SPARKLE (randomized) is ongoing, comparing the safety and efficacy of ibrutinib combined with CIT (modified RICE or RVICI) compared with CIT alone, the results of which will be published separately.

**End points and assessments**

Primary end points of part 1 of the trial included exposure (i.e., AUC), apparent (oral) plasma clearance, apparent (oral) volume of distribution, and derived measures of exposure such as maximum observed concentration, and the relationship between pharmacokinetic parameters and age or a measure of body size.

Population pharmacokinetic analysis was performed using a sparse sampling approach. Blood samples were collected predose and 1, 2, 4, and 6 hours postdose on day 1 and day 7 or 8 of cycle 1 and day 1 of cycle 2 or 3 for the measurement of plasma concentrations of ibrutinib and the PCI-45227 metabolite [2]. The two-compartment population pharmacokinetic model derived for adults was applied to the available concentration data [3]. Nonlinear mixed effects were introduced to account for both interindividual and interoccasion variability, thus allowing estimates of relevant pharmacokinetic parameters for each profile observed at each visit per patient. A minimal number of empirical or naïve covariates was introduced at the population level until the model provided satisfactory individual fits without significant correlation between random effects and the covariates of age, weight, and visit number. Pharmacokinetic parameters were derived using NONMEM version 7.4.1 (ICON, Dublin, Ireland).

Safety was a secondary end point and included monitoring of adverse events (AEs; using the National Cancer Institute Common Terminology Criteria for Adverse Events version 4.03), AEs of special interest, and clinical laboratory tests. Treatment-emergent AEs (TEAEs) related to the study drug were those determined by the investigator to be possibly, probably, or very likely related to the study drug based on the clinical scenario for individual patients. The AE of special interest was major hemorrhage, which was defined as any treatment-emergent hemorrhagic AE of grade ≥ 3 (any hemorrhagic AE requiring a transfusion of red blood cells was reported as grade ≥ 3), any treatment-emergent serious AE of bleeding of any grade, or any treatment-emergent CNS hemorrhage/hematoma of any grade.

Other secondary end points were overall response (complete response [CR], including biopsy-negative CR, unconfirmed CR, and PR), to assess preliminary efficacy. Tumor response and disease progression were assessed according to the International Pediatric NHL Response Criteria that previously described [4]. EFS was defined as the time interval from randomization to disease progression, lack of CR or PR after three cycles of treatment, or death, whichever occurred first. EFS was estimated as an exploratory analysis by the investigator in part 1 but is a primary objective assessed by an independent review committee in part 2 of the study.

**Statistical analysis**

Summary statistics for continuous variables included mean, standard deviation, median, minimum, and maximum values, unless otherwise specified. Categorical data were presented as frequencies and percentages.

Sample size calculation for Part 2 of the study (randomized) will be detailed in a future publication. Because Part 1 was an evaluation of safety and pharmacokinetics only, the sample size was not powered for prespecified effect size. Per protocol, evaluable pharmacokinetic data from 2 patients in each age cohort were to be collected, and at least 3 patients were to be treated with each regimen (ibrutinib plus modified RICE or ibrutinib plus RVICI) without any concerns for toxicity before enrollment into Part 2 of the study.

The safety population comprised all patients who received ≥ 1 dose of ibrutinib. The intent-to-treat population, consisting of all randomized patients, will be used in the complete efficacy analysis, once part 2 of the study is completed.

For preliminary efficacy analysis of part 1 of the study, the Kaplan-Meier method was used to estimate EFS. Statistical analyses were performed using the SAS software package (version 9.4, SAS Institute, Cary, NC).

**Data availability**

The data sharing policy of Janssen Pharmaceutical Companies of Johnson & Johnson is available at <https://www.janssen.com/clinical-trials/transparency>.

**Treatment-emergent hematologic abnormalities**

**Grade 3 or 4 hematologic abnormalities**

- In the ibrutinib plus modified RICE group, 9/11 (81.8%) patients had grade 3 or 4 decreases in hemoglobin, 10/11 (90.9%) had grade 3 or 4 decreases in lymphocytes, 11/11 (100.0%) had grade 3 or 4 decreases in neutrophils, and 10/11 (90.9%) had grade 3 or 4 decreases in platelets.
- In the ibrutinib plus RVICI group, 7/10 patients (70.0%) had grade 3 or 4 decreases in hemoglobin, whereas grade 3 or 4 decreases in lymphocytes, neutrophils, and platelets occurred in all patients (100.0%).

**References**

1. Swerdlow SH, Campo E, Pileri SA, Harris NL, Stein H, Siebert R *et al.* The 2016 revision of the World Health Organization classification of lymphoid neoplasms. *Blood* 2016; **127**(20): 2375–2390.

2. de Vries R, Huang M, Bode N, Jejurkar P, de Jong J, Sukbuntherng J *et al.* Bioanalysis of ibrutinib and its active metabolite in human plasma: selectivity issue, impact assessment and resolution. *Bioanalysis* 2015; **7**(20): 2713–2724.

3. Marostica E, Sukbuntherng J, Loury D, de Jong J, Woot de Trixhe X, Vermeulen A *et al.* Population pharmacokinetic model of ibrutinib, a Bruton tyrosine kinase inhibitor, in patients with B cell malignancies. *Cancer Chemother Pharmacol* 2015; **75**(1): 111–121.

4. Sandlund JT, Guillerman RP, Perkins SL, Pinkerton CR, Rosolen A, Patte C *et al.* International pediatric non-Hodgkin lymphoma response criteria. *J Clin Oncol* 2015; **33**(18): 2106–2111.

**Supplementary Table 1** Dose and administration of ibrutinib plus RICE

|  | Day | | | | | | | | | |
| --- | --- | --- | --- | --- | --- | --- | --- | --- | --- | --- |
|  | 1 | 2 | 3 | 4 | 5 | 6 | 7 | 8 | 9 | 10–28^a^ |
| Ibrutinib 329 mg/m^2,b^ | X^c^ | X | X | X | X | X | X | X | X | X |
| Rituximab 375 mg/m^2^ | X |  | X |  |  |  |  |  |  |  |
| Ifosfamide 3 g/m^2^ over 2 hours^c^ |  |  | X | X | X |  |  |  |  |  |
| Carboplatin 635 mg/m^2^ over 1 hour |  |  | X |  |  |  |  |  |  |  |
| Etoposide 100 mg/m^2^ over 1 hour |  |  | X | X | X |  |  |  |  |  |
| Mesna 600 mg/m^2^ with ifosfamide and at 3, 6, 9, and 12 hours after the start of each dose^c^ |  |  | X | X | X |  |  |  |  |  |
| Mesna 3000 mg/m^2^/day (shortened infusion)^d^ |  |  | X | X | X | X | X | X | X |  |
| Dexamethasone 20 mg/m^2^/day (3 divided doses; IV or PO) |  |  |  |  | X | X | X | X | X |  |
| IV hydration 3,000 mL/m^2^/day | X | X | X | X | X | X | X | X | X |  |
| Triple intrathecal therapy^e^ | X |  |  |  | X^e^ |  |  |  | X^e^ |  |

^a^If count recovery occurs quickly, cycles could be shortened to a minimum of 21 days.

^b^Up to a maximum dose of 560 mg/day.

^c^Ibrutinib administered early in the day. To avoid a drug-drug interaction, mesna infusion was stopped 3 hours before administering ibrutinib and not started for 6 hours after administering ibrutinib. Since mesna was given first with ifosfamide, ifosfamide dosing was separated accordingly from ibrutinib.

^d^Mesna administered as a shortened infusion instead of intermittent dosing only in patients with hematuria. To avoid a drug-drug interaction, mesna infusion was stopped 3 hours before administering ibrutinib and not started for 6 hours after administering ibrutinib.

^e^If CNS-negative, triple intrathecal therapy (methotrexate, corticosteroid [hydrocortisone or prednisolone], and cytarabine) was administered on day 1 of each cycle. If CNS-positive, triple intrathecal therapy was administered every 4 ± 1 days until cerebrospinal fluid was cleared of blasts. Intrathecal rituximab was not permitted. Intrathecal therapy was not administered more than 24 hours before day 1 of each cycle.

CNS, central nervous system; IV, intravenous; PO, orally; RICE, rituximab, ifosfamide, carboplatin, etoposide, and dexamethasone.

**Supplementary Table 2** Dose and administration of ibrutinib plus RVICI

|  | Day | | | | | | | | | | |
| --- | --- | --- | --- | --- | --- | --- | --- | --- | --- | --- | --- |
|  | 1 | 2 | 3 | 4 | 5 | 6 | 7 | 8 | 9 | 10–28^a^ |  |
| Ibrutinib 329 mg/m^2,b^ | X^c^ | X | X | X | X | X | X | X | X | X |  |
| Rituximab 375 mg/m^2^ | X |  | X |  |  |  |  |  |  |  |  |
| Vincristine 0.4 mg/m^2^/24 hour continuous  infusion over 4 days |  |  | X | X | X | X |  |  |  |  |  |
| Idarubicin 10 mg/m2 over 4 hours |  |  | X | X |  |  |  |  |  |  |  |
| Carboplatin 200 mg/m^2^/24 hours continuous  infusion over 4 days |  |  | X | X | X | X |  |  |  |  |  |
| Ifosfamide 2 g/m^2^/24 hours over 5 days  (shortened infusion)^c^ |  |  | X | X | X | X | X |  |  |  |  |
| Mesna 500 mg/m^2^ bolus |  |  | X |  |  |  |  |  |  |  |  |
| Mesna 3000 mg/m^2^/day (shortened infusion)^c^ |  |  | X | X | X | X | X | X | X |  |  |
| Dexamethasone 20 mg/m^2^/day (3 divided doses; IV or PO) |  |  |  |  | X | X | X | X | X |  |  |
| IV hydration 3000 mL/m^2^/day | X | X | X | X | X | X | X | X | X |  |  |
| Triple intrathecal therapy^d^ | X |  |  |  | X |  |  |  | X |  |  |
| GCSF |  |  |  |  |  |  |  |  |  | X^e^ |  |

^a^If count recovery occurs quickly, cycles could be shortened to a minimum of 21 days.

^b^Up to a maximum dose of 560 mg/day.

^c^Ibrutinib administered early in the day. To avoid a drug-drug interaction, mesna infusion was stopped 3 hours before administering ibrutinib and not started for 6 hours after administering ibrutinib. Since mesna was given first with ifosfamide, ifosfamide dosing was separated accordingly from ibrutinib.

^d^If CNS-negative, triple intrathecal therapy (methotrexate, corticosteroid [hydrocortisone or prednisolone], and cytarabine) was administered on the first day of each cycle. If CNS-positive, triple intrathecal therapy was administered every 4 ± 1 days until cerebrospinal fluid was cleared of blasts. Intrathecal rituximab was not permitted. Intrathecal therapy was not administered more than 24 hours before day 1 of each cycle.

^e^GCSF could be administered beginning day 12 and continued until ANC ≥ 500/µL on 2 occasions post-nadir.

ANC, absolute neutrophil count; CNS, central nervous system; GCSF, granulocyte colony stimulating factor; IV, intravenous; PO, orally; RVICI, rituximab, vincristine, ifosfamide, carboplatin, idarubicin, and dexamethasone.

**Supplementary Table 3** Grade ≥ 3 treatment-emergent adverse events that occurred in
≥ 15 % of patients in either treatment group^a^

| *n* (%) | Ibrutinib plus  modified RICE  (*n* = 11) | Ibrutinib plus RVICI  (*n* = 10) |
| --- | --- | --- |
| Blood and lymphatic system disorders | 11 (100.0) | 10 (100.0) |
| Anemia | 7 (63.6) | 8 (80.0) |
| Thrombocytopenia | 7 (63.6) | 8 (80.0) |
| Neutropenia | 6 (54.5) | 7 (70.0) |
| Febrile neutropenia | 8 (72.7) | 5 (50.0) |
| Leukopenia | 3 (27.3) | 1 (10.0) |
| Gastrointestinal disorders | 1 (9.1) | 7 (70.0) |
| Diarrhea | 0 | 4 (40.0) |
| Infections and infestations | 6 (54.5) | 8 (80.0) |
| Sepsis | 2 (18.2) | 4 (40.0) |
| Herpes zoster | 0 | 2 (20.0) |
| Device related infection | 2 (18.2) | 1 (10.0) |
| Investigations | 7 (63.6) | 5 (50.0) |
| Neutrophil count decreased | 1 (9.1) | 2 (20.0) |
| Platelet count increased | 5 (45.5) | 2 (20.0) |
| White blood cell count decreased | 1 (9.1) | 2 (20.0) |
| Alanine aminotransferase increased | 3 (27.3) | 0 |
| Aspartate aminotransferase increased | 2 (18.2) | 0 |
| Metabolism and nutrition disorders | 3 (27.3) | 7 (70.0) |
| Hypokalemia | 2 (18.2) | 5 (50.0) |
| Decreased appetite | 0 | 2 (20.0) |
| Hypophosphatemia | 1 (9.1) | 2 (20.0) |
| General disorders and administration site conditions | 1 (9.1) | 4 (40.0) |
| Mucosal inflammation | 0 | 2 (20.0) |
| Nervous system disorders | 0 | 5 (50.0) |
| Vascular disorders | 0 | 3 (30.0) |
| Hypotension | 0 | 2 (20.0) |
| Renal and urinary disorders | 2 (18.2) | 1 (10.0) |

^a^Listed by Medical Dictionary for Regulatory Activities (MedDRA) System Organ Class (Version 20.1) and Preferred Term.

Modified RICE, rituximab, ifosfamide, carboplatin, etoposide, and dexamethasone; RVICI, vincristine, ifosfamide, carboplatin, idarubicin, and dexamethasone.

**Supplementary Table 4** Grade ≥ 3 treatment-emergent adverse events related to ibrutinib^a^

| *n* (%) | Ibrutinib plus  modified RICE  (*n* = 11) | Ibrutinib plus RVICI  (*n* = 10) |
| --- | --- | --- |
| Blood and lymphatic system disorders | 5 (45.5) | 6 (60.0) |
| Neutropenia | 2 (18.2) | 6 (60.0) |
| Thrombocytopenia | 4 (36.4) | 5 (50.0) |
| Anemia | 4 (36.4) | 3 (30.0) |
| Febrile neutropenia | 3 (27.3) | 1 (10.0) |
| Leukopenia | 2 (18.2) | 0 |
| Gastrointestinal disorders | 0 | 4 (40.0) |
| Investigations | 5 (45.5) | 4 (40.0) |
| White blood cell count decreased | 1 (9.1) | 1 (10.0) |
| Alanine aminotransferase increased | 2 (18.2) | 0 |
| Platelet count decreased | 3 (27.3) | 0 |
| General disorders and administration site conditions | 0 | 3 (30.0) |
| Mucosal inflammation | 0 | 2 (20.0) |
| Metabolism and nutrition disorders | 1 (9.1) | 2 (20.0) |
| Hypokalemia | 0 | 2 (20.0) |
| Hypophosphatemia | 1 (9.1) | 1 (10.0) |
| Infections and infestations | 2 (18.2) | 1 (10.0) |
| Sepsis | 1 (9.1) | 1 (10.0) |
| Fungal | 0 | 0 |

^a^Listed by Medical Dictionary for Regulatory Activities (MedDRA) System Organ Class (Version 20.1) and Preferred Term.

Modified RICE, rituximab, ifosfamide, carboplatin, etoposide, and dexamethasone; RVICI, vincristine, ifosfamide, carboplatin, idarubicin, and dexamethasone.

**Supplementary Table 5** Treatment-emergent major hemorrhage events^a,b^

| *n* (%) | Ibrutinib plus  modified RICE  (*n* = 11) | Ibrutinib plus RVICI  (*n* = 10) |
| --- | --- | --- |
| Any treatment-emergent major hemorrhage events | 2 (18.2) | 3 (30.0) |
| Hemorrhage intracranial | 0 | 1 (10.0) |
| Intestinal hemorrhage | 0 | 1^c^ (10.0) |
| Melena | 1 (9.1) | 1^c^ (10.0) |
| Spinal cord hematoma | 0 | 1^d^ (10.0) |
| Subarachnoid hemorrhage | 0 | 1^d^ (10.0) |
| Cystitis hemorrhagic | 1 (9.1) | 0 |

^a^By preferred term and maximum toxicity grade.

^b^All major hemorrhage-related adverse events were grade 3/4.

^c^In the same patient.

^d^In the same patient.

Modified RICE, rituximab, ifosfamide, carboplatin, etoposide, and dexamethasone; RVICI, vincristine, ifosfamide, carboplatin, idarubicin, and dexamethasone.

**Supplementary Table 6** Response to treatment by histology

| Response | Histology | Ibrutinib plus modified RICE | Ibrutinib plus RVICI | Total |
| --- | --- | --- | --- | --- |
| CR/CRu | Burkitt leukemia | 1 | 2 | 3 |
|  | Burkitt lymphoma | 1 | 0 | 1 |
|  | Diffuse large B-cell lymphoma | 1 | 0 | 1 |
| PR | Burkitt leukemia | 0 | 1 | 1 |
|  | Burkitt lymphoma | 1 | 0 | 1 |
|  | Burkitt-like lymphoma | 2 | 0 | 2 |
|  | Diffuse large B-cell lymphoma | 2 | 0 | 2 |
|  | High-grade B-cell lymphoma | 0 | 1 | 1 |
| MR | Burkitt lymphoma | 1 | 1 | 2 |
|  | Burkitt-like lymphoma | 0 | 1 | 1 |
| NR | Burkitt leukemia | 1 | 0 | 1 |
|  | Burkitt lymphoma | 1 | 1 | 2 |
| NE^a^ | Burkitt leukemia | 0 | 1 | 1 |
|  | Burkitt lymphoma | 0 | 1 | 1 |
|  | B-primary mediastinal lymphoma | 0 | 1 | 1 |

^a^Patients did not complete ≥ 1 cycle of treatment.

CR, complete response; CR_u_, complete response unconfirmed; modified RICE, rituximab, ifosfamide, carboplatin, etoposide modified with dexamethasone; MR, minor response; NR, no response; NE, not evaluable, as per the international pediatric non-Hodgkin lymphoma response criteria [1]; PR, partial response; RVICI, rituximab, vincristine, ifosfamide, carboplatin, idarubicin, and dexamethasone.

1. Sandlund JT, Guillerman RP, Perkins SL, Pinkerton CR, Rosolen A, Patte C *et al*. International pediatric non-Hodgkin lymphoma response criteria. *J Clin Oncol* 2015; **33**(18): 2106–2111.

**Supplementary Figure Legends**

**Supplementary Fig. 1** Patient disposition

HSCT, hematopoietic stem cell transplantation; modified RICE, rituximab, ifosfamide, carboplatin, etoposide modified with dexamethasone; PD, progressive disease; RVICI, rituximab, vincristine, ifosfamide, carboplatin, idarubicin.

**Supplementary Fig. 2** EFS in the ibrutinib plus modified RICE and ibrutinib plus RVICI groups with a median 18-month follow-up.

EFS, event-free survival; modified RICE, rituximab, ifosfamide, carboplatin, and etoposide, with the addition of dexamethasone; RVICI, vincristine, ifosfamide, carboplatin, idarubicin, that includes dexamethasone.
