# Supplementary figures and images for: Ibrutinib plus CIT for R/R mature B-NHL in children (SPARKLE trial): initial safety, pharmacokinetics, and efficacy
Source: Leukemia. 2020 Feb 18;34(8):2271–5. doi: 10.1038/s41375-020-0749-5 (PMC7387295; doi:10.1038/s41375-020-0749-5)

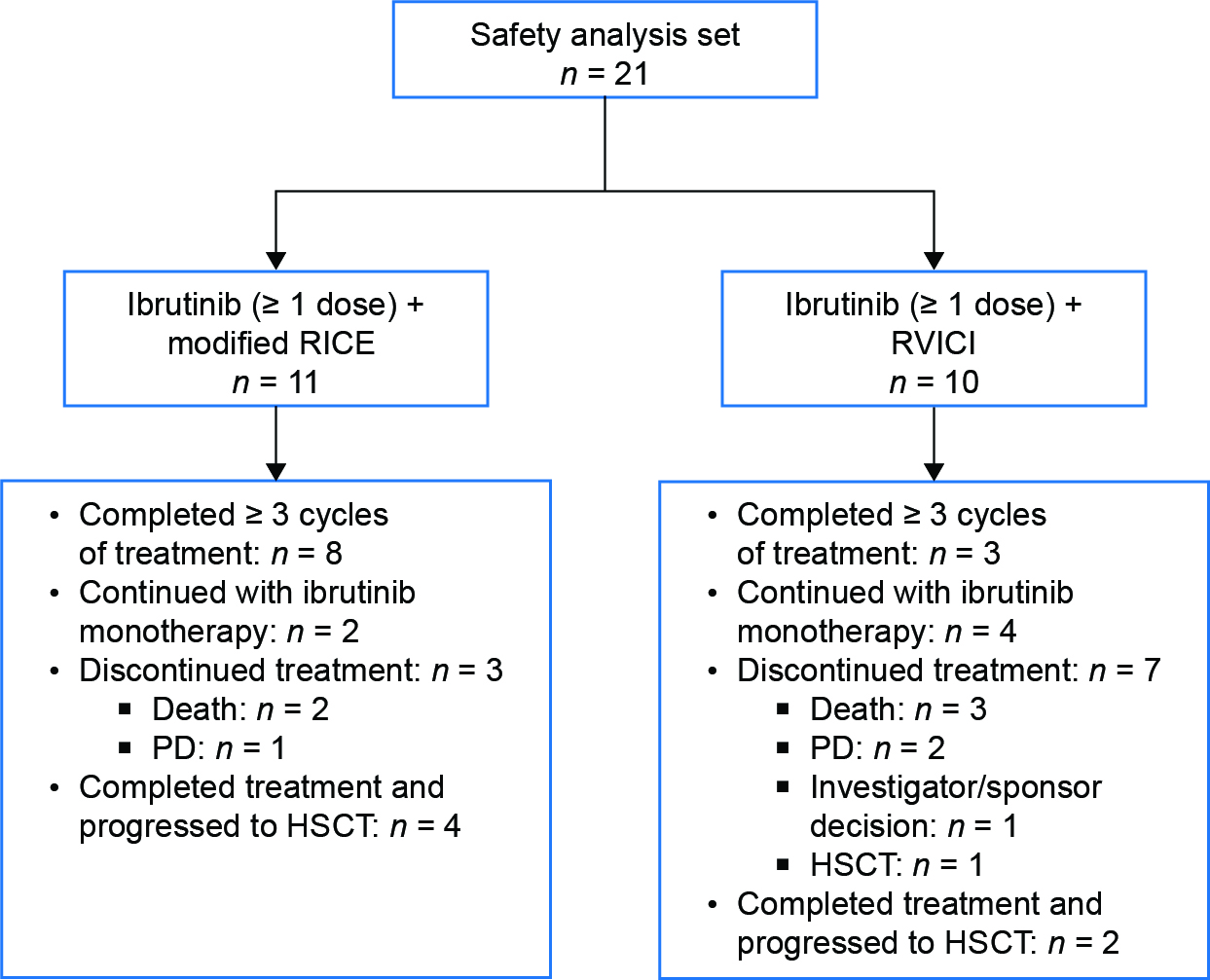

Supplement: Supplementary file 2 — Supplementary Figure 1 [file 41375_2020_749_MOESM2_ESM.jpg]
